# A comparative study of clinical manifestations, haematological and serological responses after experimental infection with *Anaplasma phagocytophilum *in two Norwegian sheep breeds

**DOI:** 10.1186/1751-0147-53-8

**Published:** 2011-02-11

**Authors:** Snorre Stuen, Lise Grøva, Erik G Granquist, Karin Sandstedt, Ingrid Olesen, Håvard Steinshamn

**Affiliations:** 1 Norwegian School of Veterinary Science, Department of Production Animal Clinical Sciences, Sandnes, Norway; 2 Bioforsk, Norwegian Institute for Agricultural and Environmental Research, Organic Food and Farming Division, Tingvoll, Norway; 3 National Veterinary Institute, Uppsala, Sweden; 4 Nofima Marin, Ås, Norway

## Abstract

**Background:**

It has been questioned if the old native Norwegian sheep breed, Old Norse Sheep (also called Norwegian Feral Sheep), normally distributed on coastal areas where ticks are abundant, is more protected against tick-borne infections than other Norwegian breeds due to a continuously high selection pressure on pasture. The aim of the present study was to test this hypothesis in an experimental infection study.

**Methods:**

Five-months-old lambs of two Norwegian sheep breeds, Norwegian White (NW) sheep and Old Norse (ON) sheep, were experimentally infected with a 16S rRNA genetic variant of *Anaplasma phagocytophilum *(similar to GenBank accession number M73220). The experiment was repeated for two subsequent years, 2008 and 2009, with the use of 16 lambs of each breed annually. Ten lambs of each breed were inoculated intravenously each year with 0.4 ml *A. phagocytophilum*-infected blood containing approximately 0.5 × 10^6 ^infected neutrophils/ml. Six lambs of each breed were used as uninfected controls. Half of the primary inoculated lambs in each breed were re-challenged with the same infectious dose at nine (2008) and twelve (2009) weeks after the first challenge. The clinical, haematological and serological responses to *A. phagocytophilum *infection were compared in the two sheep breeds.

**Results:**

The present study indicates a difference in fever response and infection rate between breeds of Norwegian sheep after experimental infection with *A. phagocytophilum.*

**Conclusion:**

Although clinical response seems to be less in ON-lambs compared to NW-lambs, further studies including more animals are needed to evaluate if the ON-breed is more protected against tick-borne infections than other Norwegian breeds.

## Background

Tick-borne fever (TBF) caused by the bacteria *Anaplasma phagocytophilum *(formerly *Ehrlichia phagocytophila*) is an endemic disease of sheep in tick (*Ixodes ricinus*) infested areas of Norway. Natural infection with *A. phagocytophilum *has been reported in humans and a variety of domestic and wild animal species [1]. TBF is seldom fatal, unless complicated by secondary infections. However, TBF causes immunosuppression, leaving sheep vulnerable to secondary infections, such as tick pyaemia caused by *Staphylococcus *spp. [2], and *Pasteurella *(*Mannheimia*) septicaemia [3,4]. Complications also include abortion in pregnant ewes [5], reduced milk yield in cattle [6], impaired spermatogenesis in rams [7], and reduced weight gain in lambs [8]. The infection in sheep may cause considerably animal welfare problems and has for decades been one of the main scourges for the Norwegian sheep industry [8].

A serological survey in sheep indicated that *A. phagocytophilum *infection is widespread along the coast of southern Norway. However, clinical TBF was only diagnosed in half of these seropositive sheep flocks [9]. The reason for this diagnostic deficit may be attributed to the existence of genetic variants of the agent causing different clinical symptoms and immunological reactions [2,10]. Based on a 16S rRNA gene sequence study, it has recently been shown that genotypes of *A. phagocytophilum *may co-exist in the same sheep flock and even in the same animal [11]. The geographical distribution of these variants is however unknown.

The management of sheep flocks may vary considerably in Norway. While the dominant Norwegian sheep breed, Norwegian White (NW) sheep, are normally housed indoors during the winter season and treated regularly against ticks and gastro-intestinal parasites, the Old Norse (ON) sheep breed can be on pasture the whole year around with limited parasitic treatment [12]. A British study indicated earlier, that there may be a difference in breed susceptibility to *A. phagocytophilum *infection [13]. Based on a continuous high selection pressure and possible also breed differences, it has been hypothesized that the ON-breed is more protected against tick-borne infections than other Norwegian breeds. The aim of the present study was therefore to test this hypothesis by experimental infection, to compare the clinical, haematological and serological responses to *A. phagocytophilum *infection in two Norwegian sheep breeds.

## Materials and methods

### Source of *Anaplasma phagocytophilum *and DNA sequencing

Blood samples were collected from a flock of Norwegian sheep known to be infected with *A. phagocytophilum*. Based on partial sequencing of the 16S rRNA gene, a variant of *A. phagocytophilum *was found in one lamb, similar to GenBank accession number M73220[11]. Both EDTA and heparinised blood samples were taken from the infected lamb. The EDTA blood samples were used to measure haematological values and to prepare blood smears. The absolute number of infected cells per unit volume was determined by multiplying the total number of neutrophils per unit volume by the percentage of infected neutrophils counted on a May-Grünwald Giemsa stained blood smear. The heparinised blood was stored at -70°C in 5 ml aliquots with 10% dimethyl sulphoxide (DMSO) as cryoprotectant without any propagation in cell culture or sequence passage through other sheep.

### Animals, experimental design, and haematology

A total number of 64 lambs, 5 months old, were used in this trial, 32 lambs of the NW-breed and 32 of the ON-breed. The experiment was approved by the National Animal Research Authority (Norway). The study was conducted for two following years, 2008 and 2009, with the yearly use of 16 lambs of each breed. The ON-lambs came from two different sheep herds in Rogaland county, lambs from one herd were used each year, while all NW-lambs belonged to the experimental sheep flock at the Department of Production Animal Clinical Sciences. All ON-lambs were adapted and housed at the department three to four months before start of the experimental period. None of the lambs had previously been exposed to pasture with *I. ricinus *and were kept indoors during the whole experimental period of four to five months. The lambs of each breed were grouped in infected and controls, according to equal distribution of sex and mean live weight. Ten lambs of each breed were inoculated intravenously (day 0) with 0.4 ml of the above mentioned DMSO-stabilate of *A. phagocytophilum *using one aliquot for each breed. The infectious blood contained approximately 0.5 × 10^6 ^infected neutrophilic granulocytes/ml. Six lambs in each group were left as uninfected controls. Nine weeks (2008) and twelve weeks (2009) after the primary inoculation, five of the earlier inoculated lambs of each breed were selected by random sampling and re-challenged with the same infectious dose of the homologous variant.

The lambs were observed at least twice a day. Rectal temperature was measured daily in all lambs throughout the experiment. The incubation period was defined as the period between inoculation and the first day of fever (≥ 40.0°C), and the duration of fever was recorded as the number of days with elevated body temperature (≥ 40.0°C). The magnitude of fever of each lamb was estimated from the area of plots of daily temperature on 5 mm grids and calculated according to the Trapezium Rule [14]. For this purpose 40°C represented the baseline.

Daily EDTA blood samples were collected during the fever period, and on a weekly basis after the fever had subsided. In addition, EDTA blood samples were later collected from individual lambs when intermittent rectal temperatures above 40.0°C were recorded. Total and differential leucocyte counts were determined electronically (ADVIA^®^, Bayer) and blood smears were prepared and stained with May-Grünwald Giemsa. Four hundred neutrophils were examined on each smear by light microscopy, and the number of cells containing *Anaplasma *inclusions was recorded. The infection rate (percentage of infected neutrophils) was calculated. The body weight of all lambs was measured weekly, during the whole experimental period. The average live weights (± SD) of the lambs at the start of the experiment were 52.8 ± 6.86 (NW-2008), 44.9 ± 6.10 (NW-2009), 28.8 ± 4.10 (ON-2008), and 21.3 ± 3.44 (ON-2009), respectively.

#### Extraction of DNA and real time PCR for the identification of *A. phagocytophilum *positive samples, targeting *msp2 *(*p44*)

In order to investigate *A. phagocytophilum *infection of non-reactive lambs, the ON-lambs (2009) were analysed for *Anaplasma *-DNA by PCR [15]. Briefly, an automated isolation procedure based on magnetic bead technology was performed by the application of the MagNA Pure LC instrument (Roche) and the MagNA Pure LC DNA Isolation Kit I Blood Cells High Performance (Roche). EDTA blood samples from the inoculated animals were thawed at room temperature and 200 μl blood was transferred to the DNA isolation procedure according to the instruction manual (Roche). The isolated DNA was eluted with 100 μl low salt buffer and stored at -20°C awaiting PCR analysis. The concentration of DNA in each sample was determined by OD_260 _spectrophotometry (GeneQuant II, Pharmacia Biotech, Cambridge, UK). The samples were diluted 1:100 before PCR analysis.

The primers were Ap MSP2 252 5' ACAGTCCAGCGTTTAGCAAGA and Ap MSP2 459 5' CACCACCAATACCATAACCA, amplifying a product of 208 bp at the N-terminal hyper variable region of the *msp2(p44) *expression site. The primers were manufactured by TIB Molbiol (Germany). A Light Cycler 480 instrument (Roche) was used for the real-time PCR analysis. A total of 96 well white plates were loaded with a reaction mix consisting of 0.5 μl (5 μM) ApMSP2 252 primer, 0.5 μl (5 μM) ApMSP2 459 primer, 1.5 μl RNAse free H_2_O, 5 μl LightCycler 480 DNA SYBR Green I Master and 2.5 μl sample. Plates were sealed by sealing foil and centrifuged at 1200 rpm for two minutes. Samples and non-template controls were run in duplicates on each plate. The C_q _values (quantification cycle) were determined by the 2^nd ^derivative maximum method and verified by melting point analysis (Tm) [15].

### Serology

Sera were collected at days 0, 7, 14, 21, 28, 42, and 63 (only first challenge) after each inoculation and analysed using an indirect immunofluorescence antibody assay (IFA) to determine the antibody titre to *Ehrlichia equi *[9,16]. Briefly, two-fold dilutions of sera were added to slides precoated with *A. phagocytophilum *(formerly *E. equi*) antigen (Protatec, St. Paul, Minn.). Bound antibodies were visualized by fluorescein-isothiocyanate (FITC)-conjugated rabbit-anti-sheep immunoglobulin (Cappel, Organon Teknika, West Chester, PA). Sera were screened for antibodies at dilution 1:40. If positive, the serum was further diluted and retested. A titre of 1.6 (log_10 _reciprocal of 1:40) or more was regarded positive.

### Statistics

Statistical calculations were done using Statistix, version 4.0 (Analytical Software), and a two-sample *t *test was used to analyse clinical, haematological and serological variables. A *P *value of < 0.05 was considered significant.

## Results

### Clinical parameters, haematology, PCR-detection and serology

In general, a one to two days period with reduced appetite was observed in the NW-lambs infected with *A. phagocytophilum*, while infected ON-lambs showed no signs of clinical illness.

**Figure 1:**
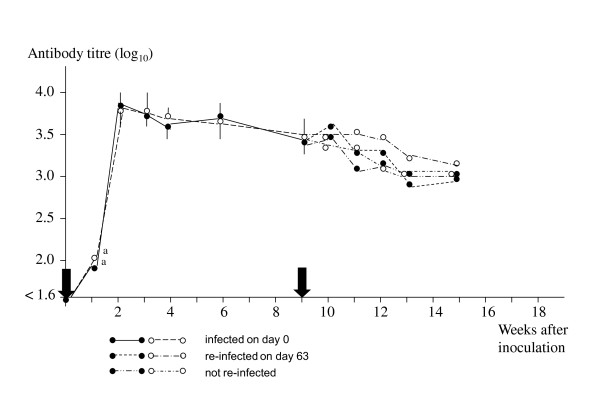
**Mean antibody titre (+SD) to *Anaplasma phagocytophilum *in lambs from two Norwegian sheep breeds, NW and ON, respectively, experimentally infected with a variant of *A. phagocytophilum *in 2008**. Ten lambs in each group were inoculated on day 0 and five lambs were re-challenged with the homologous variant after nine weeks. A titre below 1:40 (log_10 _= 1.6) was considered negative. Arrows indicate time of inoculation. ● - NW, ○ - ON, ^a ^one lamb

In 2008, all inoculated lambs from both groups developed fever. Significant differences in duration of fever and magnitude of fever were recorded between the two sheep breeds (Table 1). In addition, infection rate was significantly different on days 3, 6, 7 and 8 (Table 2). However, no significant difference was observed in the serological response for the first 63 days (Figure 1). Recurrent fever periods of one to two days duration, were observed in five (NW) and four (ON) lambs, respectively.

**Table 1:** Mean and standard deviation (± SD) of different clinical variables in 5-month-old lambs of two Norwegian Sheep breeds, Norwegian White (NW) and Old Norse (ON), respectively, infected with one variant of *A. phagocytophilum*

	**2008**			**2009**		
	
	**NW**	**ON**	**P-value**	**NW**	**ON**^**#**^	**P-value**
**Incubation period (days)**	3.0 ± 0.0	2.9 ± 0.3	ns	3.0 ± 0.0	9.6 ± 2.4	p < 0.0001
**Max. temp. (°C)**	41.87 ± 0.18	41.64 ± 0.11	ns	41.86 ± 0.13	40.96 ± 0.54	p < 0.001
**Duration of fever (days)**	8.9 ± 2.77	5.6 ± 1.02	p < 0.01	8.0 ± 3.07	5.4 ± 3.11	Ns
**Magnitude of fever (mm**^**2**^**)**	912 ± 158	503 ± 89	p < 0.001	737 ± 231	275 ± 174	p < 0.001
**Nadir of neutropenia (<0.7 × 10**^**9 **^**litre**^**-1**^**)**	0.22 ± 0.06	0.22 ± 0.08	ns	0.26 ± 0.10	0.28 ± 0.15	Ns
**Duration of neutropenia (days)**	9.1 ± 2.38	10.1 ± 2.21	ns	6.7 ± 2.24	5.7 ± 2.49	Ns
**Max. infection rate (%)**	49.0 ± 3.46	44.6 ± 5.66	ns	52.9 ± 1.79	46.6 ± 9.76	Ns
**Weight loss (%)**^**a**^	- 10.7 ± 3.21	-9.5 ± 4.63	ns	-9.6 ± 2.81	1.4 ± 4.20	p < 0.001

^a ^weight loss was measured one week after inoculation; ns - not significant

^# ^only seven lambs were infected

The study was performed in two subsequent years, 2008 and 2009, respectively. Ten lambs in each group were inoculated. A two-sample *t *test was used for statistical analysis

**Table 2:** Mean percentage of infected neutrophils observed in groups of ten lambs^$ ^of two different breeds of Norwegian sheep, Norwegian White (NW) and Old Norse (ON), respectively, inoculated with one variant of *A. phagocytophilum*

Sheep breeds				Days after inoculation/challenge							
	3	4	5	6	7	8	10	11	12	13	17
NW - 2008	43.2^#^	45.4	36.4	41.2^#^	37.8^##^	32.0^#^	22.6	3.3	<1	-	-
ON - 2008	35.1	44.4	35.8	32.8	25.0	11.7	2.1	1.9	<1		
NW - 2009	7.9	57.4	45.2	39.8	35.2	27.6	3.3	<1	-		
ON - 2009	-	-	-	-	13.0^a^	26.0^b^	47.5^b^	24.5^c^	32.1^d^	22.9^d^	3.8^b^

^$ ^only seven ON-2009 lambs were infected

Two-sample *t *test (only 2008): p < 0.05^#^, p < 0.001^##^, ^a^3 lambs, ^b^4 lambs, ^c^6 lambs, ^d^7 lambs

The study was performed in two subsequent years, 2008 and 2009, respectively

After re-challenge on day 63 (2008), clinical signs were not observed, and only one lamb of each breed reacted with a detectable infection rate (Table 3). No significant difference in the antibody titre was observed in the challenged and unchallenged lambs in either of the two breeds (data not shown).

**Table 3:** Mean percentage of infected neutrophils observed in groups of five lambs inoculated with one variant of *A. phagocytophilum *nine weeks (NW/ON-2008) and twelve weeks (NW/ON-2009) after the first inoculation, respectively

Sheep breeds			Days after inoculation/challenge					
	3	4	5	6	7	8	10	11
NW - 2008	-	<1	-	-	-	-	-	-
NW - 2009	-	-	-	-	-	-	-	-
ON - 2008	-	-	10^a^	24 ^a^	16 ^a^	1 ^a^	-	-
ON - 2009	13.5^b^	30.6	25.4	20.8	11.5^c^	5.5 ^b^	<1 ^a^	-

^a ^1 lamb, ^b ^2 lambs, ^c ^4 lambs

**Figure 2:**
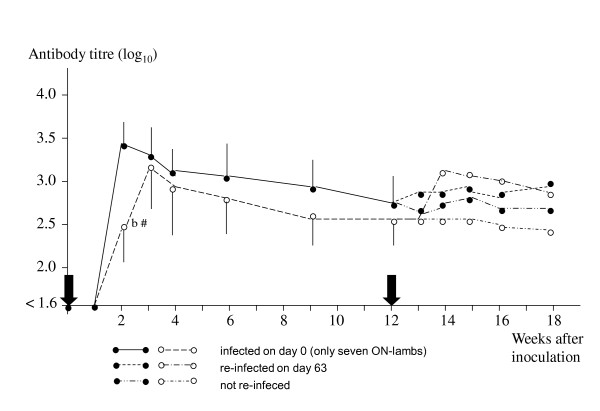
**Mean antibody titre (+SD) to *Anaplasma phagocytophilum *in lambs from two Norwegian sheep breeds, NW and ON, respectively, experimentally infected with a variant of *A. phagocytophilum *in 2009**. Ten lambs in each group were inoculated on day 0 and five lambs were re-challenged with the homologous variant after twelve weeks. A titre below 1:40 (log_10 _= 1.6) was considered negative. Arrows indicate time of inoculation. ● - NW, ○ - ON, ^b ^four lambs, Two-sample *t *test: ^# ^p < 0.05

In contrast, only seven of the infected ON-lambs in 2009 reacted with fever after the primary inoculation (Table 1). The most remarkable finding was that the incubation time in these seven ON-lambs varied from six to thirteen days (mean ± SD: 9.6 ± 2.44) and the fever period lasted for one to ten days (mean ± SD: 5.4 ± 3.11). Significant differences in incubation period, maximum temperature, magnitude of fever and weight gain were recorded between the two sheep breeds (Table 1). Two infected ON-lambs had fever for one day, with a max. temperature of 40.0°C and 40.4°C, respectively. The infection rate was displaced by several days and therefore difficult to compare from day to day (Table 3). However, the max. infection rate was not significantly different (Table 1). In addition, no significant difference between the two breeds was observed in the serological reaction for the first 84 days, except for day 14 (p < 0.05) (Figure 2).

The three ON-lambs that did not show any clinical reaction after *A. phagocytophilum *inoculation were also found negative by blood smear examination and serology. In addition, real time PCR for identification of *A. phagocytophilum *positive samples was negative in these three lambs on days 3-15 (Table 4). In the infected ON-lambs, *Anaplasma*-DNA was detected as early as five days before inclusions were observed by blood smear microscopy. Recurrent fever periods of one to two days duration, were observed in two lambs of each breed, respectively.

**Table 4:** Detection of *A. phagocytophilum *infection by real-time PCR, targeting msp-2(p44) in ON-lambs

*ON-lambs 2009*	*Day 0*	*Day 3*	*Day 5*	*Day7*	*Day10*	*Day15*
1	-	-	**+**	**+**	**+**^**#**^	**+**^**#**^
2	-	-	-	nd	**+**	**+**^**#**^
3	-	-	**+**	**+**^**#**^	**+**^**#**^	**+**^**#**^
4	-	-	-	-	-	-
5	-	-	-	nd	**+**^**#**^	**+**^**#**^
6	-	-	-	-	-	-
7	-	-	**+**	**+**	**+**^**#**^	**+**^**#**^
8	-	-	-	-	-	**+**^**#**^
9^##^	-	-	-	-	-	-
10	-	-	-	**+**^**#**^	**+**^**#**^	**+**^**#**^

^_ ^negative, + positive, nd - not done

^# ^positive by light microscopy

^## ^this lamb became infected with *A. phagocytophilum *only after re-challenge on day 84

All lambs were inoculated experimentally with *A. phagocytophilum *infected blood on day 0

After re-challenge on day 84, all five ON-lambs reacted with a clinical response. One of the lambs, which did not react after the first inoculation, now responded with a typical TBF- infection, i.e. incubation period: 3 days, max. temperature: 40.9°C; magnitude of fever: 273 mm^2 ^and duration of fever: 7 days. In addition, blood smears displayed cytoplasmic inclusions (max. infection rate: 60%), duration of neutropenia (7 days) and a serological response (titre value on day 98: 1/1280). All the other four ON-lambs also reacted with fever of 1-2 days duration, cytoplasmic inclusions (max. infection rate: 6-56%), and neutropenia (duration: 3-5 days) after re-challenge. A significant increase in antibody titre (p < 0.05) was observed in the re-challenged lambs compared with the unchallenged lambs on days 98, 105 and 112, respectively (data not shown). In contrast, the five NW-lambs that were re-challenged on day 84, did not react with a clinical response, cytoplasmic inclusions or a titre increase (Table 3, Figure 2). Clinical symptoms, haematological reaction or seroconversion were not detected in the control lambs during the experimental period.

Comparison of results from 2008 and 2009, showed no difference in clinical signs or haematological reaction between the NW-lambs (two-sample *t *test). The infection rate was significantly different only on days 3 and 4 (p < 0.001). However, significant differences were observed in the antibody titres on days 14, 21, 28, 42 and 63 (p < 0.001). Significant differences in the antibody responses were also observed between the ON-lambs in 2008 and 2009, i.e. on days 14, 21, 28, 42 and 63 (p < 0.01), respectively. In addition, variation in clinical and haematological responses and infection rates were observed within the ON-breed, such as in the maximum temperature (p < 0.05), magnitude of fever (p < 0.01), duration of neutropenia (p < 0.01) and weight gain (p < 0.001) (two-sample *t *test).

### Weight gain

In 2008, the mean daily weight gains (± SD) for the first nine weeks in the infected and control lambs were 180 ± 21.2 g/d (NW- infected), 204 ± 35.9 g/d (NW-control), 155 ± 47.8 g/d (ON-infected), and 164 ± 44.5 g/d (ON-control), respectively. No significant differences between the infected and control lambs within each breed were observed.

Similarly, the mean daily weight gains (± SD) for the first twelve weeks in the infected and control lambs in 2009 were 137 ± 30.0 g/d (NW-infected), 165 ± 16.1 (NW-control), 78.1 ± 29.0 g/d (ON-infected), and 105 ± 18.9 (ON-control), respectively. No significant differences between the infected and control lambs within each breed were observed.

## Discussion

In the present study, only a limited number of animals of each breed were included. Earlier experimental studies in NW-lambs indicate a variation in clinical, serological and haematological reactions to an *A. phagocytophilum *infection [8]. In order to evaluate the susceptibility in outbred sheep breeds, more animals should have been included. In addition, the study should have been conducted within one year to exclude annual variation in experimental conditions. However, due to limited number of lambs available, housing facilities and other practical reasons these criteria were difficult to fulfil.

Significant differences in the clinical reaction and infection rate to a primary *A. phagocytophilum *infection were detected in the two sheep breeds. In 2008, no differences in the haematological and the serological reaction were observed. In contrast, marked differences were observed in 2009, where seven ON-lambs reacted with a prolonged incubation period and no evidence of clinical response in three lambs. Breed differences in response to *A. phagocytophilum *have, as mentioned earlier, been shown among British sheep breeds. Blackface sheep reacted less severely to tick-borne fever than other breeds and their crosses, however this seems not entirely attributable to past ancestral exposure to the pathogen [13]. The reason and implication of these breed differences need further elucidation.

A significant difference in the antibody titre response was recorded between the two years. This may be due to different batches of antigen used, since the sensitivity of the IFA-test may vary between batches (Sandstedt K, personal information). The sensitivity of the present antibody test may have been increased by use of a more proper antigen, i.e. an ovine variant of the bacterium. Strong serological cross-reactions between all members of the *A. phagocytophilum *group have been reported, but the titre response to a heterologous variant is normally less than against a homologous variant [17]. Unfortunately, *E. equi *was the only antigen available for use in the present study.

In contrast to NW-lambs, marked differences in clinical and haematological reactions were observed between years in the ON-lambs. The reason for these differences among ON-lambs is unknown. The animals were from two different geographical areas with no direct relationship, and the lambs had been adapted to the same experimental conditions for several months. In addition, earlier studies indicate that the individual diversity is not significantly different within the two sheep breeds involved [18].

In 2009, seven ON-lambs reacted with a long incubation period, a low maximum temperature and a short duration of fever [8]. These reactions may indicate an innate resistance to *A. phagocytophilum*. However, an innate immune response in infected lambs may be inefficient since *A. phagocytophilum *have lost all genes for synthesis of LPS and most genes for biosynthesis of peptidoglycans [19,20].

Earlier studies indicate that the amplitude of clinical and haematological reaction is independent of the dose of the *A. phagocytophilum *inoculum, however, the incubation period may be longer with a low infection dose. As little as one *A. phagocytophilum *infected cell is enough to transmit the infection [21]. In the present study, the inoculation dose was approximately 0.2 × 10^6 ^infected neutrophils, which should be more than sufficient to create a clinical reaction. However, reduction of the infectious dose in the aliquot used during freezing and thawing cannot be excluded.

Most of the inoculated blood used in 2009 must have been infectious. However, if a small infection dose in the thawed aliquot was divided into ten infectious doses, some of these batches may have lacked infectivity. This statement is supported by the fact that *Anaplasma*-DNA was not detected in the three non-responsive lambs during the first fourteen days. The detection threshold of real-time PCR used was 10 copies of DNA [15]. These three lambs were also confirmed seronegative. In addition, when one of these three lambs was challenged on day 84, it was fully susceptible to the infection. Further studies are needed to elucidate the reason for the delayed onset of clinical reactions in ON-lambs, experimentally infected with *A. phagocytophilum*.

In the present study, the daily weight gain in lambs varied between the two years. This variation may be due to the quality of the roughage involved. Unfortunately, the quality of the silage used was not measured. The daily weight gain between the infected and non-infected animals of each breed was not significantly different. One study indicates that an early *A. phagocytophilum *infection will have a negative effect on the autumn live weight of lambs [22]. However, in that study the infected lambs were grazing on pasture, while in the present trial the experimental lambs were housed indoors under favourable environmental conditions.

Resistance to experimental re-infections rises with increasing frequency of challenge [23]. Earlier experimental studies have shown that the immunity after primary *A. phagocytophilum *infection varies and that sheep may resist homologous challenge for a period from a few months to more than one year [2,24-26]. In the present study, only the ON-lambs (2009) reacted with clinical symptoms, detectable infection rate and an increase in antibody titre after re-infection, indicating that they were not fully protected after the primary infection. The implications of relapses of clinical symptoms and bacteraemia during the persistent period are unknown. The effects of re-infections are usually less severe than the primary reaction. However, it depends on the variants of *A. phagocytophilum *involved [10].

Earlier investigations have shown that lambs are susceptible to secondary infections, especially in the bacteraemic and neutropenic periods of TBF [14,27-29]. In the present study, only the rate of bacteraemia is different between the two breeds. To evaluate if *A. phagocytophilum *infected ON-lambs are more resistant to other infectious agents compared to NW-lambs needs further investigation, since factors such as management and other external stress factors may be important to the outcome of infection [30].

## Conclusion

The present results indicate a difference in fever response and infection rate between breeds of Norwegian sheep after experimental infection with *A. phagocytophilum*. Whether these differences are due to genetic resistance that can be implemented in a breeding programme to increase the resistance against TBF, needs further exploration. However, studies including more animals are needed to evaluate if the ON-breed is more protected against tick-borne infections than the NW-breed. In addition, analyses of diseased and fatal cases of TBF must be performed in order to elucidate differences in pathogenicity and protective immunity between Norwegian sheep breeds.

## Competing interests

The authors declare that they have no competing interests.

## Authors' contributions

SS, LS, IO and HS have designed the experimental study. SS performed the experimental study, carried out the statistical analysis and drafted the manuscript. EGG carried out the molecular genetic analysis. KS performed the serology. All authors read and approved the final manuscript.
